# Characterisation of an area of coexistent visceral and cutaneous leishmaniasis transmission in the State of Piauí, Brazil

**DOI:** 10.1590/0074-02760230181

**Published:** 2024-02-05

**Authors:** Silvia Alcântara Vasconcelos, Raimundo Leoberto Torres de Sousa, Enéas Costa, João Paulo Diniz e Souza, Diane Cavalcante, Antônio Carlos Lima da Silva, Ivete Lopes de Mendonça, Jacenir Mallet, Clarissa Romero Teixeira, Guilherme Loureiro Werneck, Thais Araújo-Pereira, Daniela Pita-Pereira, Constança Britto, Maurício Luiz Vilela, Regis Gomes

**Affiliations:** 1 Fundação Oswaldo Cruz-Fiocruz, Escritório Técnico Regional, Teresina, PI, Brasil; 2 Fundação Oswaldo Cruz-Fiocruz, Instituto Oswaldo Cruz, Programa de Pós-Graduação em Medicina Tropical, Rio de Janeiro, RJ, Brasil; 3 Universidade Federal do Ceará, Departamento de Patologia, Fortaleza, CE, Brasil; 4 Fundação Nacional de Saúde, Coordenação Regional do Piauí, Núcleo de Entomologia, Teresina, PI, Brasil; 5 Universidade Federal do Piauí, Teresina, PI, Brasil; 6 Fundação Oswaldo Cruz-Fiocruz, Instituto Oswaldo Cruz, Laboratório Interdisciplinar em Vigilância Entomológica em Diptera e Hemiptera, Rio de Janeiro, RJ, Brasil; 7 Fundação Oswaldo Cruz-Fiocruz, Escritório Técnico Regional, Eusébio, CE, Brasil; 8 Universidade do Estado do Rio de Janeiro, Departamento de Epidemiologia, Rio de Janeiro, RJ, Brasil; 9 Fundação Oswaldo Cruz-Fiocruz, Instituto Oswaldo Cruz, Laboratório de Biologia Molecular e Doenças Endêmicas, Rio de Janeiro, RJ, Brasil; 10 Centro Universitário Lusíada, Santos, SP, Brasil

**Keywords:** sand flies, vector-borne disease, cutaneous leishmaniasis, natural infection

## Abstract

**BACKGROUND:**

In Brazil, transmission of visceral and cutaneous leishmaniasis has expanded geographically over the last decades, with both clinical forms occurring simultaneously in the same area.

**OBJECTIVES:**

This study characterised the clinical, spatial, and temporal distribution, and performed entomological surveillance and natural infection analysis of a leishmaniasis-endemic area.

**METHODS:**

In order to characterise the risk of leishmaniasis transmission in Altos, Piauí, we described the clinical and socio-demographic variables and the spatial and temporal distribution of cases of American visceral leishmaniasis (AVL) and American cutaneous leishmaniasis (ACL) cases and identified potential phlebotomine vectors.

**FINDINGS:**

The urban area concentrated almost 54% of ACL and 86.8% of AVL cases. The temporal and spatial distribution of AVL and ACL cases in Altos show a reduction in the number of risk areas, but the presence of permanent disease transmission foci is observed especially in the urban area. 3,808 phlebotomine specimens were captured, with *Lutzomyia longipalpis* as the most frequent species (98.45%). Of the 35 females assessed for natural infection, one specimen of *Lu. longipalpis* tested positive for the presence of *Leishmania infantum* and *Leishmania braziliensis* DNA.

**MAIN CONCLUSION:**

Our results indicate the presence of risk areas for ACL and AVL in the municipality of Altos and highlight the importance of entomological surveillance to further understand a possible role of *Lu. longipalpis* in ACL transmission.

Leishmaniasis are worldwide distributed anthropozoonosis caused by protozoan parasites of the *Leishmania* genus, with high prevalence in the Americas. One of the the six most relevant neglected diseases in the world, leishmaniasis are a group of diseases caused by *Leishmania* protozoan parasites that usually manifests as two distinct clinical forms: cutaneous leishmaniasis (CL), and visceral leishmaniasis (VL).

In Brazil, an increasing distribution and urbanisation have been observed in all regions of the country but still lack effective prevention and control policies for interrupting transmission.^1^ ^,^ ^2^ ^,^ ^3^ Global climate changes in addition to the emergence of new and complex epidemiological scenarios resulting from human intervention of the environment, have contributed to dissemination to new areas.^4^ ^,^ ^5^

In Piauí State, located in the northeast of Brazil, American VL (AVL) is endemic and was documented for the first time in 1934. Environmental changes combined with migratory flows, resulted in a process of disease urbanisation.^6^ ^,^ ^7^ Figueiredo et al.^8^ demonstrated that habitations in urban areas surrounded by vegetation had higher number of seropositive dogs for *Leishmania infantum* infection and the proximity to places with denser vegetation favours the interaction between the wild and peridomestic parasite transmission cycles and provides better conditions for the maintenance of sand fly populations, the vector of *Leishmania* parasites.

The occurrence of American CL (ACL) outbreaks has been recently reported, with the identification of species of sand flies captured by entomological surveys in distinct municipalities.^9^ ^,^ ^10^ Despite the growing number of ACL cases in Piauí, the species of *Leishmania* responsible for causing cutaneous lesions has not been identified.^11^

Batista et al.^11^ described for the first time the occurrence of ACL in Piauí, with the highest number of cases reported in 2010 and the predominant involvement of adult men, suggesting a possible occupational exposure. In 2014, there were 78 reported cases of ACL in the state. Although Brazil reported a 34% reduction in the incidence of cutaneous leishmaniasis in 2016,^12^ the municipality of Altos in Piauí State reported an increase in ACL cases in the last two years, suggesting a failure in the control strategies, the capacity of vector adaptation and increased population exposure to the risk of infection. Previous studies by our group have already demonstrated the presence of the main vectors of ACL, *Nyssomyia whitmani*, and of AVL, *Lutzomyia longipalpis*, captured in the peridomicile of both urban and rural areas of Altos.^13^ As an urban area with remained rural activity and with the constant appearance of ACL cases, the city of Altos has been chosen to further characterise the eco-epidemiology of the disease including the vectors involved, the circulating parasites and possible reservoirs.

The occurrence of the two leishmaniasis clinical forms in the same area deserves attention from the scientific community and health professionals. Epidemiological analysis and the spatial distribution of ACL and AVL cases in conjunction with the identification of sand fly vectors will contribute to the understanding of *Leishmania* spp. transmission in the municipality of Altos and may be used to guide prevention and control actions.

## MATERIALS AND METHODS

**Fig. 1:**
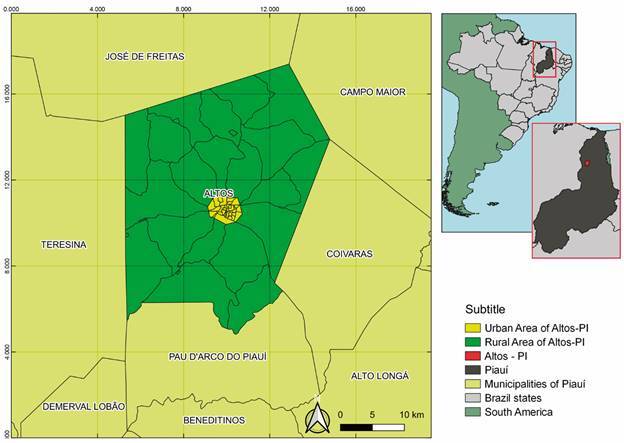
description of the municipality of Altos, Piauí State, Brazil. Map showing the location of the municipality and its urban and rural areas.

*Study design and study area* - This is a descriptive study, based on retrospectively collected secondary data on the ACL and AVL cases recorded in the municipality of Altos, in Piauí (Fig. 1). The research was implemented in the municipality of Altos, located in the Entre Rios territory, with an estimated population of 39,715 inhabitants, 957,655 km^2^ of land area and 40.54 inhabitants per km^2^. Located at an altitude of 180 m above sea level, it has an average temperature of 30ºC, with a warm and tropical climate. The average annual rainfall varies from 800 to 1600 mm, with five to six rainy months with a dry season. The months of February, March and April correspond to the wettest quarter in the region [Supplementary data (Fig. 1)].^14^

*Study population and data source* - The data on the confirmed cases of ACL and AVL from 2008 to 2018 were obtained from SINAN^15^ the disease notification system available at the Municipal Health Department of Altos and after authorisation from the ethics committee. Notification forms cover several variables, such as: sociodemographic, epidemiological, case evolution and autochthony. The variables used included date of notification, date of birth, age, gender, education, city, and district of residence, clinical manifestations, human immunodeficiency virus (HIV) co-infection, parasitological and immunological diagnosis, type of entry, initial drug choice for treatment, possible location of the infection source and case evolution. Most variables were categorical, except age, month and year of occurrence, that were continuous.

**Fig. 2:**
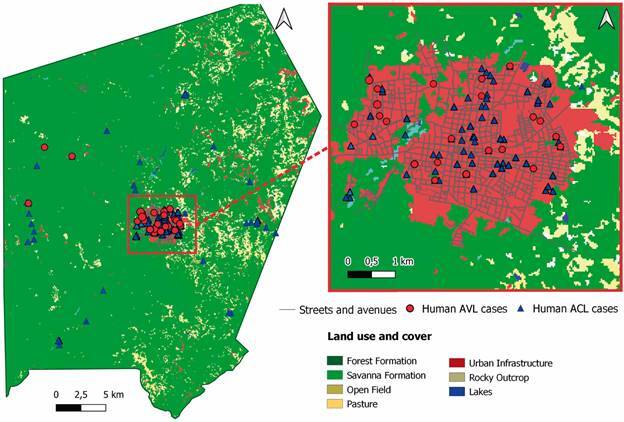
distribution of leishmaniasis cases in urban and rural areas of Altos. Map showing the localised cases of American cutaneous leishmaniasis (ACL) and American visceral leishmaniasis (AVL). The map shows the vegetation characteristics of the region and the type of soil of the studied area.

*Spatial and temporal distribution analysis* - The human cases spatial analysis was performed to define areas with higher incidence of leishmaniasis and to compare ACL and AVL distribution (Fig. 2). The addresses were geocoded using the Global Positioning System (GPS). These data were used in the construction of ACL and AVL spatial distribution maps. Kernel maps were used to assess the density distribution of cases of both diseases in the region. A comparison between the temporal distributions of ACL and AVL cases were performed using Kernel ratio maps.

*Lesions and histopathological analyses of ACL patients* - Cases of ACL in Altos were identified by physical examination and laboratory diagnosis. Two patients were followed during diagnosis and treatment and images from the lesion before and after treatment were obtained. A biopsy was performed in both patients before treatment to confirm the presence of parasites in the lesion. The tissue specimen was fixed in formalin and placed in paraffin. Histological sections were stained with HE (hematoxylin and eosin) and analysed in an optical microscope.

*Sand flies’ collection and identification* - Sand flies were collected from January/2019 to January/2020 and January/2021 to December/2021 using CDC light traps during the night, placed in the peridomicile (hen house, pigsty, and stables) and intradomicile of houses situated in urban and rural areas with high incidence of cases. According to the municipality of Altos, urban areas are characterised by a high population density, continuous presence of construction and urban infrastructure. Meanwhile, Alto’s rural zone corresponds to a set of primary activities (especially agriculture activities), practiced in areas not occupied by a large population density. Although, there is an official limit between urban and rural zones, some neighbourhoods included in the urban area (newly constructed houses and buildings) that are far from the initial urban infrastructure, are considered an urban area. We had a total of 667 hours of collection effort, five months out of the 25 months had no collection. At least two residences per neighbourhood with at least three traps, twice a week for one week per month were sampled. The geographic coordinates were obtained with a portable GPS navigator (Garmin^®^) for geocoding the homes of individuals with both AVL and ACL cases (Fig. 1). The same place was used to set the traps to capture sand flies. Climatic variables such as temperature and relative humidity were obtained using a thermo-hygrometer and rainfall data was obtained from the National Institute of Meteorology (INMET) database. Sand flies were separated and fixed in alcohol 70%, mounted on a glass slide using Berlese liquid. Identification was based on the classification method of Galati et al. and the abbreviation of genus and subgenus based on previous work.^16^ ^,^ ^17^ After morphological identification, the female’s abdomen was placed in tubes according to its species for molecular study to identify infection by *Leishmania* spp.

*Molecular detection of Leishmania DNA in sand flies and parasite species identification* - A sampling of non-engorged females was randomly selected for the individual analysis of *Leishmania* DNA detection. For this, the DNA of each sand fly was extracted and submitted to a multiplex PCR assay following a previously established protocol.^18^ The assay can simultaneously amplify the conserved region of kDNA minicircles of the *Leishmania* genus^19^ and the IVS6 region of the cacophony gene of neotropical sand flies;^20^ the latter acts as an internal control and DNA yield and purity.^18^ Male sand flies were included as negative controls for DNA extraction.

After confirming the DNA detection of *Leishmania* genus among female sand flies, the parasite species identification was performed by nested-polymerase chain reaction (PCR) amplification of the *Leishmania* hsp70 (hsp70C reverse: 5’ GGA CGA GAT CGA GCG CAT GGT 3’ and hsp70C forward: 5’ TCC TTC GAC GCC TCC TGG TTG 3’), generating a 234 bp fragment, as previously described.^21^ In the second round of amplification of the 234 bp product, the hsp70C reverse primer and a new forward primer 5’ (hsp70F2 GGA GAA CTA CGC GTA CTC GAT GAA G3’) were used, generating an internal 144 bp region of the *Leishmania* hsp70 gene.^22^ The 144 bp product was cloned using pGEM^®^-T Easy Vector Systems (Promega, Madison, USA) according to manufacturer’s recommendations. Recombinant plasmids were subjected to DNA extraction using the commercial PureLink Quick Plasmid DNA Miniprep kit (Invitrogen, Carlsbad, USA), following manufacturer’s protocol, and submitted to sequencing.

Sequencing was performed using BigDye™ Terminator v3.1 Cycle Sequencing Ready Reaction kit (Applied Biosystems, Foster City, USA), in the Sanger ABI 3730XL sequencing platform at Fiocruz-RJ. Consensus sequences were obtained and edited using the software package Phred/Phrap/Consed version: 0.020425.c (University of Washington, Seattle, USA). Sequences with Phred values above 20 were used as contigs and were assembled and aligned in MEGA5 software.^23^ Sequences were evaluated against NCBI nr database using BLASTn.

*Statistical analysis* - The chi-squared test was used to assess differences between categorical variables. Statistical significance was established when p < 0.05. For calculation of incidence, annual projections from DATASUS and IBGE (Brazilian Institute of Geography and Statistics) were used. The softwares QGIS 3.10 and TerraView were used for the geospatial analysis.

*Ethics* - The epidemiological data and addresses were obtained from the Municipal Health Department of Altos - Piauí. The data available for this research are not freely accessible. The participants are committed to data confidentiality according to the commitment term for data use. Only secondary data were used through the SINAN database preserving the identification of patients. This project was submitted to the Research Ethics Committee (CEP) of the Oswaldo Cruz Institute (IOC) and was approved under CAAE number 28217119800005248. The project was also submitted to the Biodiversity Authorisation and Information System (SISBIO), for legal authorisation to collect wild animals, approved under number 61837-1.

## RESULTS

*Socio-demographic data of ACL and AVL cases in Altos* - There was a small variation on the socio-demographic variables distribution among ACL cases in Altos (Table I). Almost no variation was observed for gender and the most affected age groups were the elderly and people between 41 and 50 years of age. The analysed group had low education, with 72.9% of reported cases with only incomplete or complete primary education, and 10.2% were illiterate. The urban area concentrated almost 54% of notified ACL cases.

**TABLE I:** Socio-demographic characteristics from patients with American cutaneous leishmaniasis (ACL) and American visceral leishmaniasis (AVL) in Altos, between 2008 and 2018

Variables	ACL (nº/%)	AVL (nº/%)	Total (nº/%)	p-value
Gender				
Male	85(51.21)	19(50.00)	104(50.98)	1.00
Female	81(48.79)	19(50.00)	100(49,02)	
Residence zone				
Urban	89(53.61)	33(84.22)	122(59.80)	< 0.001
Rural	77(46.39)	5(15.78)	82(40.20)	
Age groups (years)				
≥ 20	28(16.87)	23(60.53)	51(25.00)	< 0.001
21 - 30	17(10.24)	3(7.89)	20(9.80)	
31 - 40	24(14.46)	5(13.16)	29(14.22)	
41 - 50	35(21.08)	1(2.63)	36(17.65)	
51 - 60	26(15.66)	2(5.26)	28(13.73)	
> 60	36(21.69)	4(10.53)	40(19.60)	
Education				
Illiterate	18(10.84)	3(7.9)	21(10.29)	< 0.001
Junior school	121(72.90)	10(26.3)	131(64.22)	
High school	17(10.24)	2(5.3)	19(9.31)	
Higher education	5(3.01)	0(0)	5(2.45)	
Unknown	5(3.01)	23(60.5)	28(13.73)	

n: number.

Concerning AVL cases, there was similar numbers of men and women notified. The most affected age group was under twenty, corresponding to 60.5% of confirmed cases, followed by the group from 31 to 40 years (13.2%). Regarding education, 60.5% of cases did not give this information what compromised assessment of this indicator. The urban area presented almost all the confirmed cases, with 86.8% of the notified patients (Table I).

**Fig. 3:**
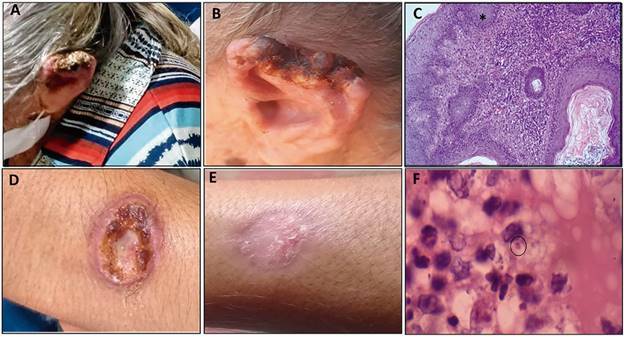
American cutaneous leishmaniasis (ACL) lesions in patients from Altos, Piauí. (A) Cutaneous lesion on the ear with intense ulceration on the extremity. (B) Extensive, nodular, ulcerated skin lesion after treatment. (C) Biopsy of the patient’s ear before treatment with epidermis exhibiting irregular acanthosis. In the dermis there is a marked lymphohistiocytic inflammatory infiltrate. (HE, 20X). (D) Single ulcerated lesion with high borders, granular base, on the patient’s forearm. (E) Appearance of the lesion after treatment. (F) Chronic inflammatory process with vacuolated cells containing an amastigote form (black circle) of *Leishmania* sp. (HE, 1000X).

*Clinical data of ACL and AVL cases in Altos* - Regarding ACL in the municipality of Altos, 97.6% of cases were classified as cutaneous lesions, with occasional occurrences of mucosal lesions. The clinical epidemiological classification criteria was the most used diagnostic method (72.3% of cases). Among the laboratory methods, Montenegro’s intradermal reaction was the most used method to confirm the disease, corresponding to 18.7% of all cases. Although few histopathological exams were performed (only 2,4%), the presence of parasites were confirmed in the ulcerated lesions from the evaluated patients. One patient presented a cutaneous lesion on the ear with severe ulceration on the extremity observed before treatment (Fig. 3A). After treatment the lesion showed an extensive, nodular, and ulcerated skin (Fig. 3B). Before treatment the epidermis exhibited irregular acanthosis and a marked lymphohistiocytic inflammatory infiltrate in the dermis (Fig. 3C). A typical ACL manifestation showing a single ulcerated lesion with elevated borders, granular centre is observed in another patient’s forearm (Fig. 3D). After treatment, the presence of a scar indicated lesion resolution (Fig. 3E). Before treatment, the lesion presented a chronic inflammatory process with vacuolated cells containing an amastigote form (black circle) of *Leishmania* spp. (Fig. 3F). The lesion histopathological findings also indicated an accentuated irregular acanthosis and the superficial and middle dermis with a marked inflammatory infiltrate. In addition, the presence of neutrophil exocytosis in the epidermal layer was observed, configuring a typical finding in lesions caused by *Leishmania* spp. [Supplementary data (Fig. 2A)]. The dermis and subcutaneous tissue contained a lymphohistiocytic inflammatory infiltrate, and granulomas containing Langerhans multinucleated giant cells [Supplementary data (Fig. 2B-C)]. The drug of first choice for treatment of most patients was the pentavalent antimony [Supplementary data (Table I)]. There was a low percentage of co-infection with HIV, only positive in two out of the 166 cases. However, the number of cases that were not tested for HIV was significant (eight cases). Regarding the therapeutic evolution, 99.4% of cases progressed to cure and there was one case of transfer. There was no record of deaths from ACL during the period investigated [Supplementary data (Table I)].

The clinical data of AVL cases show that 94.7% of registered cases were notified as new cases and there were two cases classified as recurrence. Among the clinical manifestations, the most frequent was fever (97.3%) followed by weakness, enlarged spleen, pallor, enlarged liver and weight loss. Symptoms such as jaundice, cough, oedema, infection, and haemorrhage were observed in a smaller proportion, although relevant in terms of severity and clinical outcome. The most used diagnostic method was parasitological, used in 50% of cases, followed by clinical epidemiological, with 39.5% and immunological, with 10.5%. The most used initial drug was the pentavalent antimony, in 47.4% of the cases, while amphotericin B and liposomal amphotericin B corresponding to 28.9% and 23.7% of cases, respectively. Coinfection with HIV was detected in 7.9% of cases, but it is worth mentioning the percentage of patients that did not have this information (7.9%). Although the clinical outcome was cure for the largest percentage of patients, representing 73.7%, the fatality rate from AVL was 15.8% [Supplementary data (Table II)].

**Fig. 4:**
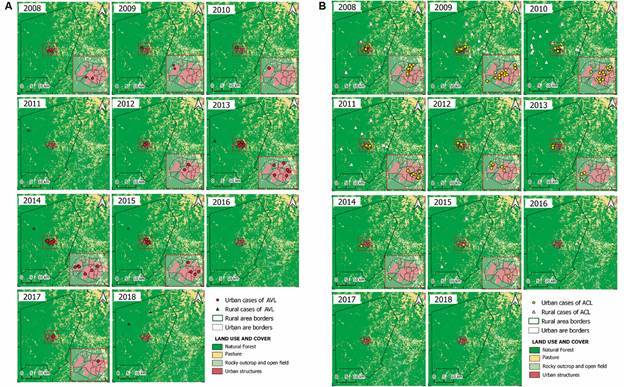
temporal distribution of leishmaniasis cases in the urban and rural areas of Altos, Piauí State, Brazil, 2008-2018. (A) AVL, American visceral leishmaniasis cases and the remote sensing of vegetation areas. (B) ACL, American cutaneous leishmaniasis cases and the remote sensing of vegetation areas.

**Fig. 5:**
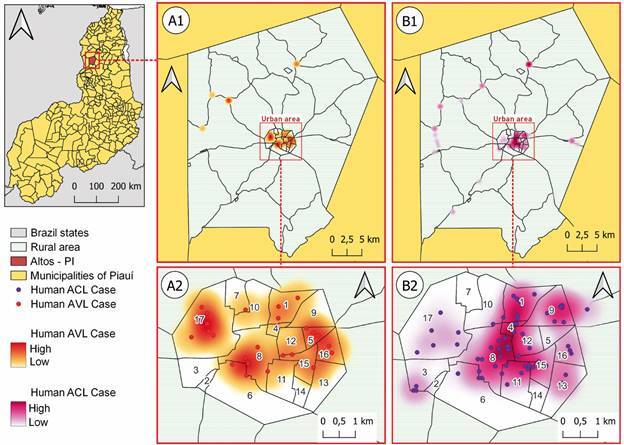
spatial distribution of leishmaniasis in the urban and rural areas of Altos, Piauí State, Brazil, 2008-2018. (A1, A2) AVL, American visceral leishmaniasis clinical forms and the density of cases. (B1, B2) ACL, American cutaneous leishmaniasis clinical forms and the density of cases. Numbers indicate the following neighbourhoods: 1. Bacurizeiro, 2. Baixão do São José, 3. Baixão dos Paivas, 4. Batalhão, 5. Boa Fé, 6. Boca de Barro, 7. Carrasco, 8. Centro 9. Ciana, 10. Leite, 11. Maravilha, 12. Matadouro, 13. Santa Inês, 14. Santo Antônio, 15. São Luiz, 16. São Sebastião, and 17. Tranqueira.

*Spatial and temporal ACL and AVL cases in Altos* - Regarding the spatial and temporal results of temporal distribution of AVL cases from 2008 to 2018, the maps indicated that there was also a heterogeneous distribution with high transmission foci in the years 2013 to 2015 (Fig. 4A). The spatial distribution of ACL cases was heterogeneous over the years. Although most areas were of low transmission intensity, there were areas with higher intensity of ACL cases, located in the northeast region of the city, from 2008 to 2012, mainly at the neighbourhoods of Ciana, Bacurizeiro, Batalhão and Centro, all located in the urban areas of Altos (Fig. 4B). The neighbourhoods of Tranqueira, Carrasco and São Luís, all located in urban areas in the northwest region of the city, presented the highest intensity of AVL reported cases over the studied years (Fig. 5A).

*Sand fly population and natural infection* - The total number of captured sand fly specimens was 3,808 (69 females and 3,739 males) composed of 98.45% of *Lu. longipalpis*, the natural vector of AVL, in a ratio of 106,11 males for each female. We also captured 56 specimens of *Ny. whitmani*, the natural vector of ACL,^24^ in a ratio of 0.64 males for each female, and three (03) male specimens of *Evandromyia lenti* (Table II). Eighty-six percent (86.3%) of the traps had captured sand flies, with positive collections in all neighbourhoods of the municipality during the almost two years of collection.

Most female sand flies were captured in the urban area of Altos. A total of 35 (20 non-engorged and 15 engorged) females were captured for evaluation of natural infection by *Leishmania* spp. [Supplementary data (Table III)].

**TABLE II:** Species of sand flies captured in the municipality of Altos / Piauí

Species	Sex n (%)			
	Male	Female	Total	Ratio
*Lutzomyia longipalpis*	3.714 (97,53)	35 (0,92)	3.749 (98,45)	106,11
*Nyssomyia whitmani*	22 (0,58)	34 (0,89)	56 (1,47)	0,64
*Evandromyia lenti*	3 (0,08)	0 (0)	3 (0,08)	0
Total	3.739 (98,19)	69 (1,81)	3.808 (100)	54,18

n: number.

Of note, one sand fly tested positive to natural infection by multiplex PCR assay, and subsequently submitted to genotyping by cloning and sequencing of the *hsp 70* gene. Sequencing results identified this female sand fly harbouring two species of parasites: *L. infantum*, the etiological agent of AVL, and *L. braziliensis*, etiological agent of ACL (Table III). Our protocol uses the kDNA as target for detecting DNA of the *Leishmania* genus in sand flies due to its high sensitivity.^18^ The *hsp 70* gene is used to genotype the parasite following a positive kDNA result. Considering that the *hsp 70* gene is present in low copy numbers in the *Leishmania* genome, it is not an ideal target for parasite DNA screening in the vector. So, for the correct detection and identification of *Leishmania* spp. in sand flies, a first detection step is necessary using PCR directed to kDNA and after, the parasite genotyping by nested-PCR of the *hsp 70* gene, followed by cloning and sequencing of an internal fragment of this gene.^25^

**TABLE III:** Detection of natural infection of sand flies by *Leishmania* spp.

Samples	Sequence	Description	Scientific name	Max score	Total score	Query cover	value	Per. ident	Accession
S - 62 clone 1	GGAGAACAACGCGTACTCGATGAAGAACACGCTCGGCGACTCGAACGTGTCCGGCAAGCTGGACGATAGCGACAAGGCCACGCTGAACAAGGAGATCGACGTGACGCTGGAGTGGCTGAGCAGCAACCAGGAGGCGTCGAAGG	*Leishmania infantum* isolate ph06 putative heat-shock protein hsp70 gene, partial cds	*Leishmania infantum*	254	254	100%	3E-63	98.60%	MF137828.1
S - 62 clone 2	AATTCGATTGGAGAACTACGCGTACTCGATGAAGAACACGGTCTCCGACACGAACGTGTCCGGCAAGCTGGAGGAGAGCGACAGGTCCGCGCTGAACTCGGCGATCGACACGGCGCTGGAGTGGCTGAACAGCAACCAGGAGGCGTCGAAGGAAAT	*Leishmania braziliensis* voucher LBCE 18678 heat shock protein 70 (HSP70) gene, partial cds	*Leishmania braziliensis*	270	270	93%	3E-68	100.00%	MN395479.1

Max: maximum; Per: percentage; Ident: identity.

**Fig. 6:**
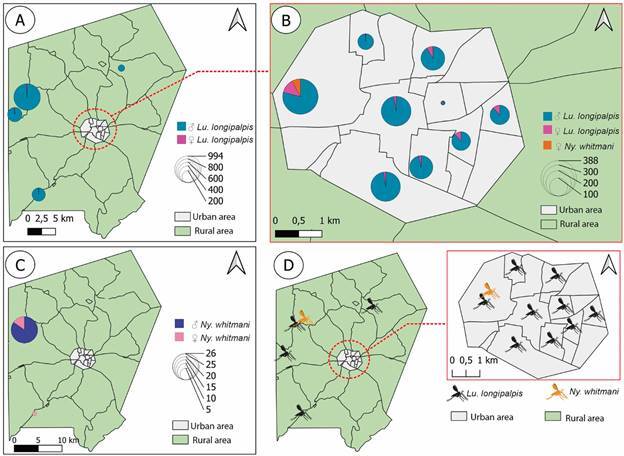
evaluation of sand flies distribution in the rural and urban areas of Altos, Piauí State, Brazil. (A) *Lutzomyia longipalpis* male prevalence in the rural area. As the circle increases, the greater is the number of sand flies captured. (B) *Lu. longiplapis* male prevalence in urban area. (C) *Nyssomyia whitmani* male prevalence in rural area. (D) Presence of both *Lu. longipalpis* and *Ny. whitmani* in the urban area with a prevalence of *Lu. longipalpis*.

Sand fly distribution in the urban area of the city, showed males and females scattered in several neighbourhoods, both at the most central and peripheral areas. More specimens of *Lu. longipalpis* were collected compared to the other species (Fig. 6). The presence of *Ny. whitmani* was detected in the peri-urban neighbourhood bordering the rural area, and rural area with closed and dense forest vegetation, characteristic of the São Bento locality (Fig. 6). Although not statistically significant, it is possible to notice a positive correlation between the number of *Ny. whitmani* males and variables such as temperature and rainfall and a negative correlation with relative humidity, demonstrating that the decrease in humidity and the increase in rainfall and temperature could influence the abundance of this species [Supplementary data (Table IV)]. Between January 2019 and January 2021, the vector density was higher at the end of the rainy season, corresponding to the months of May and June (2020), with the predominance of *Lu. longipalpis*, representing 98.45% of specimens (3749/ 3808) [Supplementary data (Fig. 1)].

**Fig. 7:**
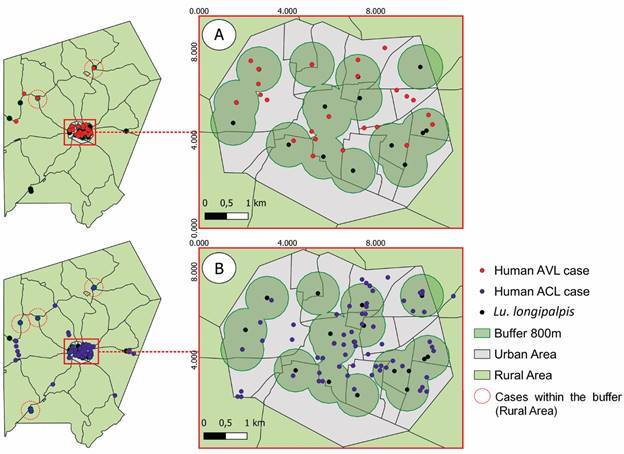
potential buffer distance of *Lutzomyia longipalpis* dispersion in the rural and urban areas of Altos, Piauí State, Brazil. Potential buffer distance of *Lu. longipalpis* dispersion in areas of (A) human visceral leishmaniasis occurrence and (B) human American cutaneous leishmaniasis (ACL) occurrence.

The dispersion distance of sand flies can vary between 200 m and 800 m, so we constructed a potential buffer distance of 800 m around the areas of positive collection points for the presence of *Lu. longipalpis*. Importantly, both ACL and AVL cases are located within the potential buffer distance of *Lu. longipalpis* dispersion (Fig. 7). This correlation between flight dispersion distance of *Lu. longipalpis* and the location of AVL cases reinforce the active transmission of AVL in the municipality of Altos. The registered cases of ACL and AVL are located within the potential flight dispersion distance of *Lu. longipalpis*.

## DISCUSSION

This study demonstrated and further characterised the transmission of AVL and ACL in Altos, a small town with an ongoing urbanisation process.^11^ Altos is the second municipality in Piauí State in the number of settlements registered in the National Institute of Colonisation and Agrarian Reform (INCRA), indicating an increased human occupation of natural areas and consequent exposure to potential vectors. The growing urbanisation process combined with disorderly occupation and predatory exploitation of the environment, in addition to limited access to treatment and lack of sanitation can contribute to the prevalence of leishmaniasis.^1^ ^,^ ^6^ ^,^ ^7^ The transmission of *Leishmania* involves an environment that promotes close interactions of different species of sand flies, hosts and reservoirs that increases with urbanisation.^6^

ACL and AVL cases in Altos indicate that the disease affects both men and women similarly. This data is in accordance with previous studies, reported in Paraná State^26^ and in the city of Barbalha, in the northeast of Brazil.^27^

The most frequent clinical manifestations of patients with AVL were fever, weakness, enlargement of the spleen and liver. These results are in accordance with a study performed in Campo Grande where fever, splenomegaly and hepatomegaly were present in 95%, 85% and 78% of AVL cases, respectively. Fever and hepatosplenomegaly were present in 69% of cases.^28^ In an endemic area for AVL in Maranhão, the most reported symptom was also fever, in 97.7% of the cases. The parasitological diagnosis with parasite visualisation was performed in 53.7% of cases.

Patients with ACL present ulcerated lesions with hyperplasia, ulceration, inflammation, and a dense and diffuse dermal infiltrate with identification of amastigote forms of the parasite, typical findings in ACL lesions.^29^ ^,^ ^30^ ^,^ ^31^ Although almost all ACL cases were characterised by an ulcer with elevated borders, it is worth emphasising the epidemiological importance of occurrence of mucocutaneous cases in Altos considering its potential for tissue destruction, deformity, and impact on the quality of life of those affected. The percentage of mucocutaneous cases was lower than the national average (5.56%) in 2018 and other endemic countries such as Spain (11%) and Nicaragua (10%).^32^ ^,^ ^33^ Late diagnosis still represents a major limitation for both ACL and AVL treatment and control. The reported cases of ACL were diagnosed based on clinical and epidemiological criteria, in contrast to other endemic areas, where laboratory methods are the reference method for disease diagnosis.^27^ ^,^ ^34^ Although we have not identified the *Leishmania* species responsible for ACL cases, we detected a vector harbouring both *L. infantum* and *L. braziliensis*, responsible for AVL and ACL cases, respectively. However, at this moment, we cannot exclude the possibility of *L. infantum* causing cutaneous lesions. Cases of cutaneous leishmaniasis caused by *L. infantum* are not common but have been previously reported in Brazil and Turkey.^35^ ^,^ ^36^ ^,^ ^37^ ^,^ ^38^

Pentavalent antimony was chosen for the initial therapy for most cases, and still represents the first choice of treatment for ACL and AVL considering the reduced therapeutic options available for this treatment.^39^ Today, miltefosine is the first line of treatment for ACL, characterised by better results regarding the outcome of cure and less toxicity compared with pentavalent antimony.^40^

The temporal and spatial distribution of AVL and ACL cases in Altos show a reduction in the number of risk areas, but the presence of permanent disease transmission foci is observed especially in the urban area. This observation highlights the epidemiological importance of control strategies.^26^ ^,^ ^34^ Determination of AVL and ACL cases clusters can provide important information to guide active surveillance and decrease transmission of both diseases. This was observed in a previous study in Acre state, Brazil. Analysis of the spatial distribution of ACL cases, showed a decreasing trend in the number of cases over the years, with high-risk clusters in six municipalities, concentrating 67% of ACL cases.^34^

Here, we show that the collected sand fly species were *Lu. longipalpis*, *Ny. whitmani* and *Ev. lenti*. Importantly, we detected the presence of DNA from two different parasites species, *L. braziliensis* and *L. infantum*, in a *Lu. longipalpis* female sand fly. Several sand fly species have been described as vectors of *L. braziliensis* and there is evidence of its susceptibility to *L. braziliensis* infection demonstrated in laboratory reared sand flies.^38^ ^,^ ^41^ *Lu. longipalpis* is responsible for the establishment of *L. infantum* in Latin America but it is also considered a permissive vector, supporting development of different *Leishmania* species under experimental conditions.^42^ ^,^ ^43^ ^,^ ^44^ The detection of *L. braziliensis’* DNA in *Lu. longipalpis* has been previously described in the South-Easten region of Brazil as well as in laboratory conditions, where 70% of *Lu. longipalpis* females developed late-stage *L. braziliensis*.^45^ ^,^ ^46^ Although our results indicate that *Lu. longipalpis* is the most abundant vector in the area we do not have enough evidence to incriminate *Lu. longipalpis* as a new vector of ACL.

In nature, the dispersion distance of sand flies can vary between 200 m and 800 m.^47^ In the municipality of Altos, we analysed the potential buffer distance of dispersion surrounding the sand fly positive collection areas with the presence of *Lu. longipalpis*. The analysed map from 2008 to 2018 showed more cases of ACL than AVL, where most of ACL cases occurred within the *Lu. longipalpis* buffer distance of dispersion, both in urban and rural areas. This information suggests that transmission of *L. braziliensis* in this region might have a participation of *Lu. longipalpis* since the number of *Ny. whitmani* captured was low. However, we cannot exclude the possibility of the low number of *Ny. whitmani* and the high male:female ratio of the species captured to be related to the field sampling methodology used and the distinct characteristics and biodiversity of the studied area.^48^ *Ny. whitmani* is known to be important for the sylvatic cycle of ACL transmission in the north of Brazil but it can invade the human habitat and adapt to spaces altered by man, frequently detected in anthropic environments in the Northeast region.^49^ ^,^ ^50^ In Altos, *Ny. whitmani* was frequently detected in rural areas but a small number could be captured in urban areas suggesting adaptation to the peridomiciliary environment.^51^ More recently, this sand fly species was also identified in Teresina, the capital of Piauí, and other regions of the state.^9^ Taken together, our results characterise the dynamics of the sand fly populations and contributed to the understanding of ACL epidemiology and transmission in an endemic area where AVL and ACL are both present. The evidence that *Lu. longipalpis* and *Ny. whitmani* coexist in this area emphasises the importance of further investigations to reinforce the detection of natural infections. Since *Lu. longipalpis* can be experimentally infected with *L. infantum* and *L. braziliensis* producing infective forms, investigation of the possibility of *L. braziliensis* transmission by *Lu. longipalpis* is critical in an area with favourable conditions for the spreading of leishmaniasis. Moreover, the establishment of an efficient method of diagnosis in the initial stages of the disease is important since leishmaniasis persistence is often related to late diagnosed cases or incomplete treatment of ACL lesions.^52^ ^,^ ^53^

The presence of ACL and AVL transmission highlights the urgency of planning and executing more active health policies for entomological surveillance, prevention, and control of leishmaniasis in the municipality.
