# Supplementary material for: Characterisation of an area of coexistent visceral and cutaneous leishmaniasis transmission in the State of Piauí, Brazil
Source: Mem Inst Oswaldo Cruz. 2024 Feb 5;119:e230181. doi: 10.1590/0074-02760230181 (PMC10841424; doi:10.1590/0074-02760230181)
Supplement: Supplementary file 1 [file 1678-8060-mioc-119-e230181-s.pdf]

TABLE I

Clinical characteristics from patients with American cutaneous leishmaniasis (ACL) in Altos, between 2008 and 2018

Variables	ATL (n°/%)
Clinical forms	
Cutaneous	162 (97,6)
Mucosal	4 (2,4)
Information type	
New case	156 (93,97)
Relapse	10 (6,03)
Transfer	0 (0)
Ignored	0 (0)
HIV coinfection	
Yes	1 (0,60)
No	158 (95,18)
Unknown	7 (4,21)
Direct parasitology	
Yes	8 (4,81)
No	5 (3,01)
Not performed	153 (92,18)
Histopathology	
Parasite	4 (2,4)
Compatible finding	4 (2,4)
Not compatible	2(1,2)
Not performed	156 (94)
MIR*	
Yes	44 (26,5)
No	0 (0)
Not performs	122 (73,5)
Epidemiological classification	
Autochthonous	164 (98,8)
Imported	0 (0)
Unknown	2 (1,2)
Classification basis	
Laboratory	44 (26,5)
Epidemiological clinic	122 (76,5)
Initial medication	
Pentavalent antimonial	162 (97,6)
Amphotericin B	2 (1,2)
Pentamidine	0 (0)
Others	2 (1,2)
Not done	0 (0)
Evolution	
Cure	165 (99,4)
Death	0 (0)
Transfer	1 (0,6)

*Montenegro intradermic reaction. Source: Municipal Health Department of Altos, Piauí

TABLE II

Clinical characteristics from patients with American visceral leishmaniasis (AVL) in Altos, between 2008 and 2018

Variables	AVL (n°/%)
Information type	
New case	36 (94,74)
Relapse	2 (5,26)
Transfer/ignored	0 (0) / 0(0)
HIV coinfection	
Yes	3 (7,9)
No	32 (84,2)
Ignored	3 (7,9)
Clinical manifestation	
Fever	37 (97,36)
Weakness	35 (92,10)
Oedema	8 (21,05)
Slimming	13 (34,21)
Cough	8 (21,05)
Pallor	29 (76,31)
Spleen enlargement	31 (81,57)
Clinical aspect	7 (18,42)
Bleeding	3 (7,9)
Liver enlargement	20 (52,63)
Jaundice	10 (26,31)
Direct parasitology	
Yes	19 (50)
No	11 (28,94)
Not performed	8 (21,05)
Immunological diagnosis	
Immunofluorescence - positive	4 (10,52)
Others - positive	19 (50)
Not performed	15 (39,47)
Initial medication	
Pentavalent antimonial	16 (42,1)
Amphotericin B/ Liposomal amphotericin B	10 (26,31) / 9 (23,68)
Pentamidine/others/not threated	0(0) /0(0) /3 (7,9)
Classification basis	
Laboratory	35 (92,10)
Epidemiological clinic	3 (7,9)
Epidemiological classification	
Autochthonous	37 (97,36)
Imported/Unknown	1 (2,63) / 0(0)
Evolution	
Cure	28 (73,68)
Death by VL	6 (15,78)
Death by others causes /Abandonment	1 (2,63) /1(2,63)
Transfer	2 (5,26)

CC BY

TABLE III

Species of sand flies captures in the municipality of Altos / Piauí according to blood fed status

	Area, blood fed status and number			
	Urban area		Rural area	
Species	Engorged female	Not engorged female	Engorged female	Not engorged female
Lutzomyia longipalpis	10	14	5	6
Nyssomyia whitmani	0	30	2	2
Evandromyia lenti	0	0	0	0
Total	10	44	7	8

TABLE IV

Spearman correlation between sand fly gender and climatic variables

		Temperature (ºC)	Relative humidity (%)	Rainfall (mm)
Lutzomyia longipalpis males	Spearman corr.	0,10475	0,02824	0,04418
	p-value	0,6603	0,90593	0,85328
Lutzomyia longipalpis females	Spearman corr.	-0,1856	0	0,34568
	p-value	0,43337	1	0,13547
Nyssomyia whitmani males	Spearman corr.	0,34255	-0,02744	0,35254
	p-value	0,13929	0,90857	0,12738
Nyssomyia whitmani females	Spearman corr.	0,17931	-0,37825	0,10428
	p-value	0,4494	0,10009	0,66173
Evandromyia lenti males	Spearman corr.	0,09961	-0,01991	-0,22942
	p-value	0,67606	0,93361	0,33056

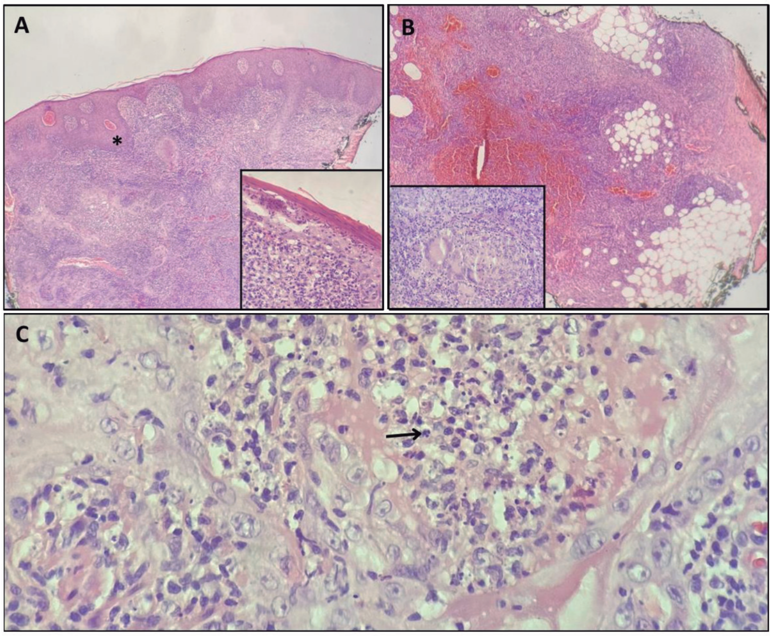

Fig. 1: histopathological aspects of the skin biopsy of lesions from patients with cutaneous tegumentary leishmaniasis. (A) Skin with irregular acanthosis (*) and superficial and middle dermis with intense inflammatory infiltrate (HE, 40X). In detail, neutrophil exocytosis in the epidermal layer (HE, 300X). (B) Middle, deep dermis and subcutaneous tissue containing lymphohistiocytic inflammatory infiltrate (HE, 40X) with granuloma formation, Langehans multinucleated giant cells (In detail. HE, 300X). (C) Lymphohistiocytic and neutrophilic inflammatory process (arrow) (HE, 400X).

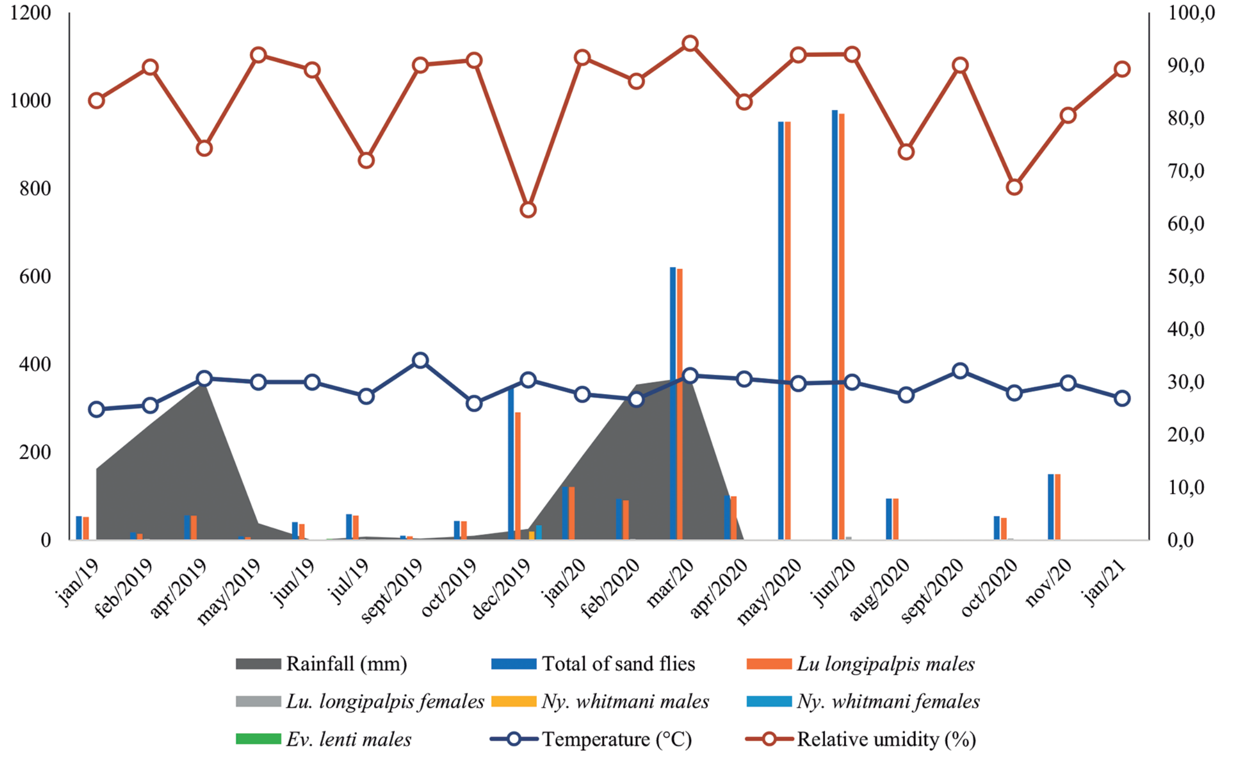

Fig. 2: number of sand fly species collected and the monthly distribution of climatic variables. Relationship between sand fly presence and the average monthly distribution of rainfall (mm), temperature (°C) and relative humidity (%).
